# Molecular Characterisation of Chikungunya Virus Infections in Trinidad and Comparison of Clinical and Laboratory Features with Dengue and Other Acute Febrile Cases

**DOI:** 10.1371/journal.pntd.0004199

**Published:** 2015-11-18

**Authors:** Nikita Sahadeo, Hamish Mohammed, Orchid M. Allicock, Albert J. Auguste, Steven G. Widen, Kimberly Badal, Krishna Pulchan, Jerome E. Foster, Scott C. Weaver, Christine V. F. Carrington

**Affiliations:** 1 Department of Preclinical Sciences, Faculty of Medical Sciences, The University of the West Indies, St. Augustine, Republic of Trinidad and Tobago; 2 Public Health England, HIV & STI Department, London, United Kingdom; 3 Institute for Human Infections and Immunity and Department of Microbiology and Immunology, University of Texas Medical Branch, Galveston, Texas, United States of America; 4 Department of Biochemistry and Molecular Biology, University of Texas Medical Branch, Galveston, Texas, United States of America; 5 Adult Emergency Department, Eric Williams Medical Sciences Complex, Mt. Hope, Republic of Trinidad and Tobago; Universidade de Sao Paulo, BRAZIL

## Abstract

Local transmission of Chikungunya virus (CHIKV) was first documented in Trinidad and Tobago (T&T) in July 2014 preceding a large epidemic. At initial presentation, it is difficult to distinguish chikungunya fever (CHIKF) from other acute undifferentiated febrile illnesses (AUFIs), including life-threatening dengue disease. We characterised and compared dengue virus (DENV) and CHIKV infections in 158 patients presenting with suspected dengue fever (DF) and CHIKF at a major hospital in T&T, and performed phylogenetic analyses on CHIKV genomic sequences recovered from 8 individuals. The characteristics of patients with and without PCR-confirmed CHIKV were compared using Pearson’s χ^*2*^ and student’s t-tests, and adjusted odds ratios (aORs) and 95% confidence intervals (CIs) were determined using logistic regression. We then compared signs and symptoms of people with RT-qPCR-confirmed CHIKV and DENV infections using the Mann-Whitney U, Pearson’s χ^*2*^ and Fisher’s exact tests. Among the 158 persons there were 8 (6%) RT-qPCR-confirmed DENV and 30 (22%) RT-qPCR-confirmed CHIKV infections. Phylogenetic analyses showed that the CHIKV strains belonged to the Asian genotype and were most closely related to a British Virgin Islands strain isolated at the beginning of the 2013/14 outbreak in the Americas. Compared to persons who were RT-qPCR-negative for CHIKV, RT-qPCR-positive individuals were significantly more likely to have joint pain (aOR: 4.52 [95% CI: 1.28–16.00]), less likely to be interviewed at a later stage of illness (days post onset of fever—aOR: 0.56 [0.40–0.78]) and had a lower white blood cell count (aOR: 0.83 [0.71–0.96]). Among the 38 patients with RT-qPCR-confirmed CHIKV or DENV, there were no significant differences in symptomatic presentation. However when individuals with serological evidence of recent DENV or CHIKV infection were included in the analyses, there were key differences in clinical presentation between CHIKF and other AUFIs including DF, which can be used to triage patients for appropriate care in the clinical setting.

## Author Summary

Chikungunya virus (CHIKV) recently emerged in the Americas and caused a major epidemic of chikungunya fever (CHIKF). While not usually life threatening, CHIKF is a debilitating and often chronic illness resulting in major morbidity and economic losses. It is difficult to distinguish CHIKF from other viral illnesses that cause acute fevers including dengue fever (DF), an important consideration since DF can be life-threatening and early identification and treatment of cases is key to reducing mortality. In this study we investigated individuals presenting to a major hospital in Trinidad and Tobago (T&T) with suspected DF or CHIKF, and identified signs and symptoms that distinguish these two illnesses. We also recovered complete genome sequences for CHIKV and show that the etiologic strain in T&T is a closely related descendent of the strain first isolated from the British Virgin Islands at the beginning of the outbreak in the Americas.

## Introduction

Chikungunya virus (CHIKV; Family *Togaviridae*, genus *Alphavirus*) is the aetiological agent of chikungunya fever (CHIKF), which presents as an acute onset high fever with headache, back pain, muscle and joint pain. The joint pain can vary in intensity but is often very intense, predominates at the ankles, wrists and phalanges and is coupled with swelling. CHIKF is usually a self-limiting disease, and serious outcomes (e.g. neurological complications) and fatalities appear to be rare [1,2]. However up to 60% of infections are followed by chronic arthritic conditions, with recurrent debilitating joint pain several years post-infection [3,4]

CHIKV exists as a single serotype thought to confer life-long immunity in recovered individuals. There is, however, sufficient variation to discern three genotypes, namely the enzootic West African (WAf), and East /Central/South African (ECSA), and the epidemic Asian genotypes. The ECSA recently gave rise to the Indian Ocean Lineage (IOL) responsible for epidemics in the Indian Ocean islands, mainland India and Europe beginning in 2004 [5]. The expansion of this lineage has been attributed to adaptive mutations in the E1 and E2 envelope glycoproteins that provide a fitness advantage in *Aedes albopictus* without reducing fitness in *A*. *aegypti* [6] and permit rapid lineage diversification [7] [8].

In light of the dramatic re-emergence of CHIKV in Asia, the intensity of global travel and the widespread prevalence of *A*. *aegypti* and *A*. *albopictus*, the emergence of CHIKV in the Americas was long anticipated [9]. As expected, numerous imported (travel-related) cases were subsequently documented in the Americas, none of which resulted in local transmission until December 2013 when autochthonous transmission was documented in the Caribbean island of St. Martin [10]. The etiologic virus, which belongs to the Asian genotype rather than the IOL [11,12], spread rapidly first amongst the islands of the Caribbean archipelago then on to the mainland Americas [13–15]. As of 7 August 2015 CHIKF cases were confirmed in 50 countries / territories in the Caribbean and mainland Americas with approximately 1.7 million suspected / confirmed cases since October 2013 [16]. CHIKV belonging to the ECSA genotype was also recently documented in Brazil but has not been confirmed elsewhere in the Americas [17].

Although CHIKF is rarely life threatening, the symptoms can be severely incapacitating, rendering patients unable to perform normal tasks or go to work. As a result, particularly in immunologically naïve populations where attack rates can be as high as 30–50% and the vast majority of infections are symptomatic [18,19] [20], the disease burden, potential to overwhelm public health systems and indirect costs can be very significant. Clinical overlap and confusion with other acute undifferentiated fevers e.g. dengue disease (which can be fatal), are additional concerns in affected regions.

The Caribbean twin-island Republic of Trinidad and Tobago (T&T) reported its first cases of CHIKV infection in July 2014 (in Trinidad) [21]. In this study we report the genetic characterisation of CHIKV and compare clinical, laboratory and epidemiological characteristics of patients with and without confirmed CHIKV or dengue virus (DENV) infections who presented at a major hospital in Trinidad, during an acute undifferentiated febrile illness (AUFI) surveillance study.

## Methods

### Study population, recruitment and sample collection

Study participants were enrolled at the Adult Primary Care Facility (APCF) of the Eric Williams Medical Sciences Complex (EWMSC) during the period of December 25^th^ 2013 to November 5^th^ 2014. Individuals were recruited, on average, 3 days a week throughout this period, with the exception of October 2014, when no sample collection was undertaken. Individuals presenting at the APCF on study days with suspected dengue fever (DF) or CHIKF were eligible for enrollment. Specifically, patients were eligible for enrollment if they were febrile (having an axillary temperature of ≥38°C) or had a reported history of fever within the last 7 days, accompanied by one or more of the following symptoms commonly associated with DF and CHIKF: headache, nausea, vomiting, diarrhoea, aches and pains, eye pain, evidence of haemorrhagic manifestation, rash, joint pain and swollen joints. Individuals who had a readily identifiable focus of infection were excluded from the study.

At presentation and enrollment, whole blood was collected in non-additive and/or ethylenediaminetetraacetic acid (EDTA) containing blood collection tubes and a questionnaire eliciting relevant demographic and clinical information, medical and recent travel history, and risk factors for exposure to mosquito bites was administered. Whole blood was stored at 4°C and centrifuged at 1500g for 10 minutes at 4°C within 24 hours. Sera and plasma samples were stored at -80°C until use.

### Reverse transcription-quantitative polymerase chain reaction (RT-qPCR) detection of CHIKV and DENV viral RNA

Nucleic acid extraction was performed using QIAamp Viral RNA Mini Kit (Qiagen; Valencia, CA) and carried out according to the manufacturer’s instructions. Extractions were performed using 140μl of serum, or plasma where serum was unavailable. Nucleic acid samples were screened and serotyped for DENV-1-4 using the DENV multiplex RT-qPCR assay previously described by Waggoner [22]. Screening for CHIKV was carried out using the RT-qPCR general assay previously described by Panning [23]. All RT-qPCR screening was done using the SuperScript III Platinum One-Step qRT-PCR kit (Invitrogen; Carlsbad, CA) with final reaction volumes amended to 25μl instead of the manufacturer’s recommended 50μl.

### Illumina sequencing

For ten samples with low Ct values in RT-qPCR assays, viral RNA was extracted then fragmented by incubation at 94°C for eight (8) minutes in 19.5 μl of fragmentation buffer (Illumina Inc., San Diego, CA). First and second strand synthesis, adapter ligation and amplification of the library were performed using the Illumina TruSeq RNA Sample Preparation kit v2 under conditions prescribed by the manufacturer (Illumina Inc., San Diego, CA). Cluster formation of the library DNA templates was performed using the TruSeq PE Cluster Kit v3 (Illumina Inc., San Diego, CA) and the Illumina cBot workstation using conditions recommended by the manufacturer. Paired end 50 base sequencing by synthesis was performed using TruSeq SBS kit v3 (Illumina Inc., San Diego, CA) on an Illumina HiSeq 1000 using protocols defined by the manufacturer.

Cluster density per lane was 820–940 k/mm^2^ and post-filter reads ranged from 148–218 million per lane. Base call conversion to sequence reads was performed using CASAVA-1.8.2. Reads were filtered for quality and adapter sequences were removed, then viral contigs were assembled *de novo* using AbySS software [24]. Assembled contigs were checked using bowtie2 to align reads to the contigs [25] followed by visualization using the integrative genomics viewer [26].

### Phylogenetic analyses

Newly generated CHIKV genomic sequences along with sequences from Genbank representing all three genotypes were aligned using the ClustalW alignment tool [27] within Geneious Version 7.1.7 (www.geneious.com; [28]). Sequences were trimmed to 11,259 nucleotides at the boundaries of the open reading frames (ORFs) so that ambiguous alignment of the 5’ and 3’ untranslated regions was excluded from analyses. The final data set comprised of 74 of these complete coding sequences from 23 countries isolated during 1953–2014 (S1 Table). Nucleotide and amino acid position numberings are based on NCBI reference sequence NC_004162.

To rule out the presence of recombination within the data set, which could affect the phylogenetic structure, all genomic sequences were screened using SBP and GARD [29] from the HyPhy online package [30]. The best-fit model for subsequent analyses (GTR+G_4_) was selected using JMODELTEST 2.1.7 [31,32]. Phylogenetic trees were inferred under this model using the maximum-likelihood (ML) method in the PhyML program online [33] and the maximum clade credibility (MCC) from BEAST version 1.8.1 [34].

Substitution rates and times to the most recent common ancestor (T_MRCA_) were jointly estimated using BEAST, with the GTR+G_4_ model of nucleotide substitution, along with a relaxed lognormal molecular clock model [35] and a GMRF skyline tree prior [36]. The analysis ran in duplicate for 50 million generations with 10% removed as burnin, and the convergence of parameters were assessed by calculating the effective sample size (ESS >200) using TRACER v1.6 [37].

### Dengue-specific IgG and IgM Enzyme-linked immunosorbent assay (ELISA)

Panbio Dengue IgM and IgG Capture ELISA (Brisbane, Australia) were used to screen sera for the presence of DENV-specific antibodies using manufacturer’s instructions, and all samples were screened in duplicate. Samples were designated as positive for dengue-specific IgM if both duplicates returned a value of >11 Panbio Units and as negative at <9 Panbio Units. Samples were designated as positive for dengue-specific IgG if both duplicates returned a value of >22 Panbio Units and as negative at <18 Panbio Units. For samples returning equivocal results (9 to 11 and 18 to 22 Panbio Units) or where the duplicates fell into different categories (i.e. one weakly positive or negative and the other equivocal), the test was repeated. Samples that remained equivocal were designated as such and for those with that remained with the duplicates falling in different categories, the mean value was taken and the sample classified accordingly.

According to the manufacturer (due to the detection thresholds for the two assays), the presence of detectable levels of IgM (in the absence of detectable IgG) indicates a probable recent primary infection with DENV, while the presence of detectable levels of IgG indicates probable recent secondary dengue virus infection. The manufacturers note that IgM levels in a secondary infection may be undetectable by the assay. Thus IgM+/IgG- result was interpreted as evidence of a probable recent primary DENV infection, and both IgM+/IgG+ and IgM-/IgG+ results were interpreted as probable recent secondary DENV infections.

### Chikungunya-specific IgM Enzyme-linked immunosorbent assay (ELISA)

Euroimmum Chikungunya IgM Capture ELISA (Leubeck, Germany) was used to screen sera and plasma for the presence of CHIKV-specific IgM antibodies. ELISAs were carried out in accordance with the manufacturer’s instructions and all samples were screened in duplicate. Samples were designated as positive for CHIKV-specific IgM if both duplicates returned a value of ≥ 1.1 and as negative at < 0.8. No sample tested returned an equivocal result (≥ 0.8 to < 1.1).

### Statistical analysis

The frequencies of patient demographic and clinical characteristics were determined by questionnaire. The outcome for analysis was having a confirmed infection with CHIKV, defined as a positive result on RT-qPCR. Bivariable associations with a confirmed CHIKV infection were determined using the student’s t-test for continuous variables and the Pearson’s chi-square or Fisher’s exact test for categorical variables. All variables with a *p*-value less than or equal to 0.10 were considered in the final multivariable model, which was determined using binary logistic regression using a forward stepwise selection procedure (entry: 5%, exit: 10%). Adjusted odds ratios (aORs) and 95% confidence intervals (CIs) are reported.

To compare the symptomatic presentation for RT-qPCR positive DENV and CHIKV, a bivariable analysis was performed using the Mann-Whitney U test for continuous variables and the Pearson’s chi-square or Fisher’s exact test for categorical variables. Data were entered using Microsoft Excel 2007 (Microsoft Corporation, Redmond, WA, USA), then transferred to IBM SPSS Statistics v20 (IBM Corporation, Armonk, NY, USA) for analysis. All *p*-values <0.05 were considered significant.

Positive predictive values (PPVs), negative predictive values (NPVs), and the sensitivity, specificity and likelihood ratios of different combinations of clinical features used to distinguish confirmed and probable DF and CHIKF from other AUFIs were also calculated. Combinations used to differentiate DF from CHIKF were also compared. For both CHIKF and DF these values were calculated using confirmed cases (RT-PCR positive for virus) and probable recent cases (ELISA positive for IgM/IgG antibodies).

### Accession numbers

The 8 newly generated CHIKV whole genome sequences are available from Genbank. Accession numbers: KR046227, KR046228, KR046229, KR046230, KR046231, KR046232, KR046233 and KR046234.

### Ethical approval

The study protocols were approved by the Ethics Committee of the University of the West Indies, St. Augustine and the Trinidad and Tobago Ministry of Health. Permission to carry out the study at the EWMSC was granted by the North Central Regional Health Authority (NCRHA). Written informed consent was obtained from all participants.

## Results

### Frequencies

There were 158 patients who met the inclusion criteria for AUFI and agreed to participate in the study. Of these, the median age was 32, ranging from 16–88 years. Half (50%) of the patients were male and, of those with complete data on ethnicity, the majority were Afro- (33%), Indo-(31%) Trinidadian or Mixed (26%) (S2 Table). Just under a third (30%) had received greater than a secondary level of education, and most (69%) were employed. Most patients (79%) were nationals of Trinidad & Tobago, with relatively few (7%) reporting a travel history outside of Trinidad in the two weeks prior to interview. Twenty-two percent reported having had laboratory-confirmed DF previously, 25% reported that others in their household had been febrile in the two weeks prior to their interview, 55% reported storing water at home and, while 15% reported having screened windows at home, most (80%) reported a history of mosquito bites at their place of residence.

**Fig 1:**
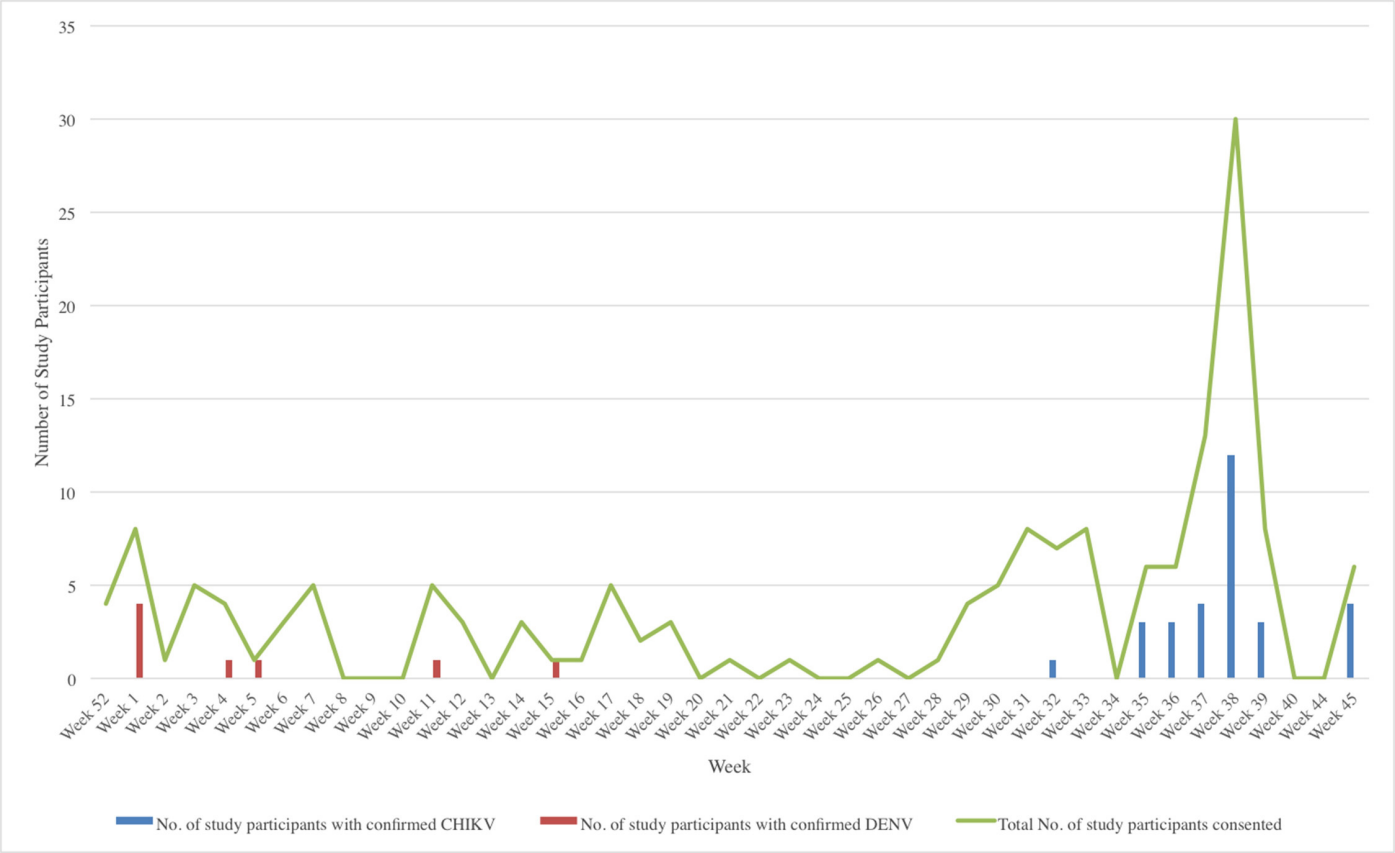
Total number of patients enrolled and number of CHIKV and DENV RT-qPCR positive cases by week. The green line graph indicates the number of patients enrolled per week. Histogram bars indicate the number of individuals confirmed as RT-qPCR positive for CHIKV (blue) or DENV (red) by RT-qPCR. Weeks 41–43 in 2014 are excluded as no patient enrolment was attempted during this period.

At interview, the most commonly reported symptoms were headache (83%), weakness (67%), joint pain (65%) and muscle pain (65%). Seventeen percent reported some degree of haemorrhagic manifestations and 22% reported a rash (S2 Table). Thirty (19%) of the 158 individuals were positive for CHIKV on RT-qPCR. The earliest case was an individual sampled on August 5^th^, 2014, about three weeks after the first CHIKV case was confirmed in Trinidad [38]. The number of RT-qPCR confirmed cases peaked in week 38 when the number of individuals enrolled was highest (Fig 1). Cases were concentrated along the urbanised “East-West corridor” in the north of Trinidad primarily within the boundaries of the North Central Regional Health Authority served by the EWMSC. Additionally, 27 (17%) of the 158 individuals were positive by ELISA for anti-CHIKV IgM antibodies, including 6 of those persons positive by RT-qPCR for CHIKV.

Eight of the 158 individuals (5.1%) were confirmed as DENV-positive by RT-qPCR (Fig 1). Of these, 6 were DENV-1 and one each DENV-3 and DENV-4. For 125 individuals (including 6 of the aforementioned RT-qPCR positive individuals), there was sufficient serum available for both dengue-specific IgM and IgG ELISAs (S3 Table). Using the combined RT-qPCR and ELISA results, 4.8% (n = 6) were designated as “confirmed current DENV infections” of which three were primary infections (i.e. IgM+/IgG-) and three were secondary infections (IgM+/IgG+), 18.4% (n = 23) were “probable recent primary DENV infections”, 42.4% (n = 53) were “probable recent secondary DENV infections” and 3.2% (n = 4) were equivocal. In total 66% (n = 82) had evidence of a current or probable recent DENV infection (S3 Table).

### Bivariable analyses

On bivariable analyses (Table 1 & S4 Table), patients with RT-qPCR confirmed CHIKV infection (CHIKV+; n = 30) were significantly more likely to report joint pain (83%, *p* = 0.020) than individuals who were RT-qPCR-negative for CHIKV (not CHIKV+) but were less likely to have travelled outside of Trinidad in the 2 weeks prior to interview (17%, *p* = 0.044), or to have reported laboratory-confirmed dengue previously (7%, *p* = 0.026). They were also more likely to have presented to the APCF earlier (mean days post onset of fever: 2.39 [CHIKV+] vs. 3.33 [not CHIKV+], *p* = 0.021) and had lower mean white blood cell counts (6.52 [CHIKV+] vs. 8.36x10^3^/μl [not CHIKV+], *p* = 0.016).

**Table 1:** Bivariable associations between select characteristics of patients of the APCF of the EWMSC, T&T (Dec 2013 –Nov 2014) and having a confirmed infection^ⱡ^ with CHIKV.

Parameters	Not confirmed		Confirmed		p-value
	n	(%)	n	(%)	
Demographics					
Age, years					
<32	64	(50.0)	11	(36.7)	0.188
>32	64	(50.0)	19	(63.3)	
Sex					
Male	68	(53.1)	11	(36.7)	0.105
Female	60	(46.9)	19	(63.3)	
Marital status					
Not married^a^	58	(47.5)	7	(26.9)	0.054†
Married (including common-law)	64	(52.5)	19	(73.1)	
Nationality					
Not Trinidadian or Tobagonian	24	(18.8)	10	(33.3)	0.080†
Trinidadian or Tobagonian	104	(81.3)	20	(66.7)	
Risk factors					
Travelled outside of Trinidad in the 2 weeks prior to interview	6	(5.0)	5	(16.7)	0.044*
Previous dengue	32	(26.0)	2	(6.9)	0.026*
Clinical factors					
Headache	108	(84.4)	23	(76.7)	0.313
Muscle pain	81	(63.3)	21	(70.0)	0.489
Joint pain	78	(60.9)	25	(83.3)	0.020*
Rash	24	(18.8)	10	(33.3)	0.080†
Sore throat	29	(22.7)	1	(3.3)	0.015*
Abdominal pain	40	(31.2)	2	(6.7)	0.006**
Any haemorrhagic manifestation^b^	23	(18.0)	3	(10.0)	0.414
	Mean	(s.d.)^‡^	Mean	(s.d.)^‡^	*p-value*
Days post onset of fever	3.33	(1.96)	2.39	(1.77)	0.021*
White blood cell count (10^3^/ml)	8.36	(4.85)	6.52	(3.25)	0.016*
Haematocrit (%)	43.81	(23.7)	39	(7.99)	0.276
Platelet count (10^3^/ml)	213.2	-100.86	234.6	(72.48)	0.277

A more comprehensive list of variables is presented in S2 Table.

^ⱡ^ Defined as a positive result by RT-qPCR

^†^ *p*<0.10

*p<0.05

**p<0.01

^‡^ Standard deviation

^a^ Includes: single, divorced and widower

^b^ Includes: nose bleed, gum bleed, abnormal vaginal bleeding, blood in urine, bruising or blood in stool

In addition to the above variables, the following also met the criteria for inclusion in the multivariable model: marital status (*p* = 0.054), nationality (*p* = 0.080), number of days post onset of illness (*p* = 0.032), rash (*p* = 0.084), sore throat (*p* = 0.015), and abdominal pain (*p* = 0.006). Due to a highly significant negative correlation between nationality and travel history, only travel history was included in the model. Similarly, due to a strong, positive and highly significant correlation between number of days post onset of fever and days post onset of illness, only the former was considered in the model. Lastly, due to small cell sizes (n ≤ 2), having had dengue previously, sore throat and abdominal pain were not considered in the final model.

### Multivariable analyses

The following variables were considered in the final multivariable model: marital status, joint pain, rash, days post onset of fever and white blood cell count. As shown in

Table 2, those who had confirmed CHIKV infection were significantly more likely to have reported joint pain (aOR: 4.52, 95% CI: 1.28–16.00), and significantly less likely to be interviewed at a later stage of illness (days post onset of fever—aOR: 0.56, 95% CI: 0.40–0.78) and have a lower white blood cell count (aOR: 0.83, 95% CI: 0.71–0.96).

**Table 2:** Multivariable associations with having a confirmed infection^ⱡ^ with CHIKV in patients of the APCF of the EWMSC, T&T (Dec 2013 –Nov 2014).

	Adjusted odds ratio	(95% confidence interval)	*p*-value
Joint pain			
No	1	Reference	
Yes	4.52	(1.28,16.00)	0.019*
Days post onset of fever	0.56	(0.40,0.78)	0.001**
White blood cell count (10^3^/μl)	0.83	(0.71,0.96)	0.014*

^ⱡ^ Defined as a positive result by RT-qPCR

*p<0.05

**p<0.01

### Comparison of patients positive by RT-qPCR for DENV and CHIKV

Among the 38 patients positive by RT-qPCR for CHIKV (n = 30) or DENV (n = 8), there were no significant differences in clinical presentations (p > 0.05) between those with CHIKV and those with DENV (Table 3 and S5 Table). However, when compared to DENV, persons with CHIKV more often reported joint pain (83% vs. 50%, *p* = 0.071) and rash (33% vs. 0%, *p* = 0.082), and less often reported abdominal pain (7% vs. 38%, *p* = 0.053). Patients with DENV had significantly lower median white blood cell count (3.50 vs. 6.00 x10^3^/μl, *p* = 0.028) and platelet (PLT) counts (1.47 vs. 2.39x10^5^/μl, *p* = 0.022).

**Table 3:** Bivariable associations between symptoms and clinical characteristics of patients of the APCF of the EWMSC, T&T (Dec 2013 –Nov 2014) and having a confirmed infection^ⱡ^ with either DENV or CHIKV.

	Status				
	DENV		CHIKV		p-value
	n	(%)	n	(%)	
Symptoms					
Headache	7	(87.5)	23	76.7)	0.66
Muscle pain	5	(62.5)	21	(70.0)	0.689
Joint pain	4	(50.0)	5	(83.3)	0.071^†^
Back pain	1	(12.5)	0	0	0.211
Rash	0	0	10	(33.3)	0.082^†^
Fatigue	1	(12.5)	15	(50.0)	0.106
Eye pain	5	(62.5)	12	(40.0)	0.426
Cough	3	(37.5)	7	(23.3)	0.411
Nausea	4	(50.0)	6	(20.0)	0.17
Vomiting	4	(50.0)	8	(26.7)	0.232
Diarrhoea	2	(25.0)	5	(16.7	0.624
Sore throat	1	(12.5)	1	(3.3)	0.381
Weakness	5	(62.5)	21	(70.0)	0.689
Stiff neck	1	(12.5)	4	(13.3)	1
Dizziness	2	(25.0)	13	(43.3)	0.44
Disorientation	0	0	3	(10.0)	1
Abdominal pain	3	(37.5)	2	(6.7)	0.053^†^
Any haemorrhagic manifestation^a^	0	0	3	(10.0)	1
	Median	Min-Max‡	Median	Min-Max‡	p-value
Days post onset of fever	2.5	1.0–5.0	2	0.0–6.0	0.484
Temperature (°C)	37	36.7–39.3	38	37.0–39.0	0.288
White blood cell count (10^3^/ml)	3.5	1.7–6.3	6	2.7–17.6	0.028*
Haematocrit (%)	43.1	37.9–48.8	39.85	12.9–51.4	0.092^†^
Platelet count (10^3^/ml)	147	51.0–294.0	239	83.0–375.0	0.022*

A more comprehensive list of variables is presented in S5 Table.

^ⱡ^ Defined as a positive result by RT-qPCR

^†^ *p*<0.10

*p<0.05

^‡^ Minimum-Maximum

^a^ Includes: nose bleed, gum bleed, abnormal vaginal bleeding, blood in urine, bruising or blood in stool.

### Distinguishing confirmed and probable recent DF and CHIKF from other AUFIs using clinical features

The clinical features joint pain and white blood cell count <7 x10^3^/μl were selected as they were significant on bivariable analysis. For DF, PLT count <150 x10^3^/ μl and abdominal pain were also selected as patients with confirmed DENV infection were more likely to display/report these symptoms than persons with confirmed CHIKV infection. Similarly for CHIKF rash was included as patients with confirmed CHIKV infection were more likely to report this sign than persons with confirmed DENV infection (Table 4).

**Table 4:** Positive predictive values (PPVs), negative predictive values (NPVs), sensitivity, specificity and likelihood ratios of clinical features used to distinguish patients of the APCF of the EWMSC, T&T (Dec 2013 –Nov 2014) with DF ^ⱡ^ and CHIKF ^ⱡ^ from patients with other AUFIs.

Parameters	PPV (%)	NPV (%)	Sens (%)	Spec (%)	Likelihood Ratio (+)	Likelihood Ratio (-)
CHIKV						
Joint pain	43.7	89.1	88.2	45.8	1.6	0.3
Rash	70.6	78.1	47.1	90.6	5.0	0.6
White blood cell count (<7x10^3^/μl)	48.8	83.6	78.0	57.7	1.8	0.4
Rash & joint pain	72.4	94.0	87.5	85.5	6.0	0.2
Rash & white blood cell count (<7 x10^3^/μl)	74.1	86.9	71.4	88.3	1.9	0.3
Joint pain & white blood cell count (<7x10^3^/μl)	20.0	96.3	83.3	56.5	2.5	0.5
Rash, joint pain & white blood cell count (<7x10^3^/μl)	77.3	96.3	94.4	83.9	5.9	0.1
DENV						
Platelet count (<150x10^3^/μl)	84.6	34.9	28.2	88.2	2.4	0.8
Abdominal pain	77.4	34.8	28.6	82.1	1.6	0.9
Abdominal pain & platelet count (<150 x10^3^/μl)	83.3	48.2	15.2	96.3	4.1	0.9

^ⱡ^ Defined as a positive result by RT-qPCR and/or positive for IgM/IgG antibodies by ELISA

Individually none of the clinical features was able to efficiently differentiate DF or CHIKF from other AUFIs. The results are given in Table 4. However the combination of rash, joint pain and white blood cell count <7 x10^3^/μl was most efficiently able to differentiate CHIKF from other AUFIs. This combination had a PPV and NPV of 77% and 96% respectively. The three parameters combined had a sensitivity of 94% and a specificity of 84%. Furthermore the positive and negative likelihood ratios were 5.9 and 0.1 respectively (Table 4).

### Distinguishing DF from CHIKF using clinical features

None of the combinations tested was overall able to efficiently differentiate between DENV and CHIKV. The combination that performed the best was rash, joint pain and white blood cell count <7 x10^3^/μl with a PPV and NPV of 58% and 95% respectively as well as a sensitivity of 88% and a specificity of 78% (Table 5).

**Table 5:** Positive predictive values (PPVs), negative predictive values (NPVs), sensitivity, specificity and likelihood ratios of clinical features used to distinguish patients of the APCF of the EWMSC, T&T (Dec 2013 –Nov 2014) with DF ^ⱡ^ infection from patients with CHIKF^ⱡ^.

Parameters	Sens (%)	Spec (%)	PPV (%)	NPV (%)
Platelet count (<150x10^3^/μl), abdominal pain	7.3	96.3	75.0	40.6
Joint pain, rash	72.7	82.1	53.3	91.4
Joint pain, white blood cell count (<7x10^3^/μl)	91.7	50.0	37.9	94.7
White blood cell count (<7x10^3^/μl), rash	50.0	84.4	53.3	82.6
White blood cell count (<7x10^3^/μl), rash, joint pain	87.5	78.3	58.3	94.7

^ⱡ^ Defined as a positive result by RT-qPCR and/or positive for IgM/IgG antibodies by ELISA

### Molecular characterisation and phylogeny of CHIKV

**Fig 2:**
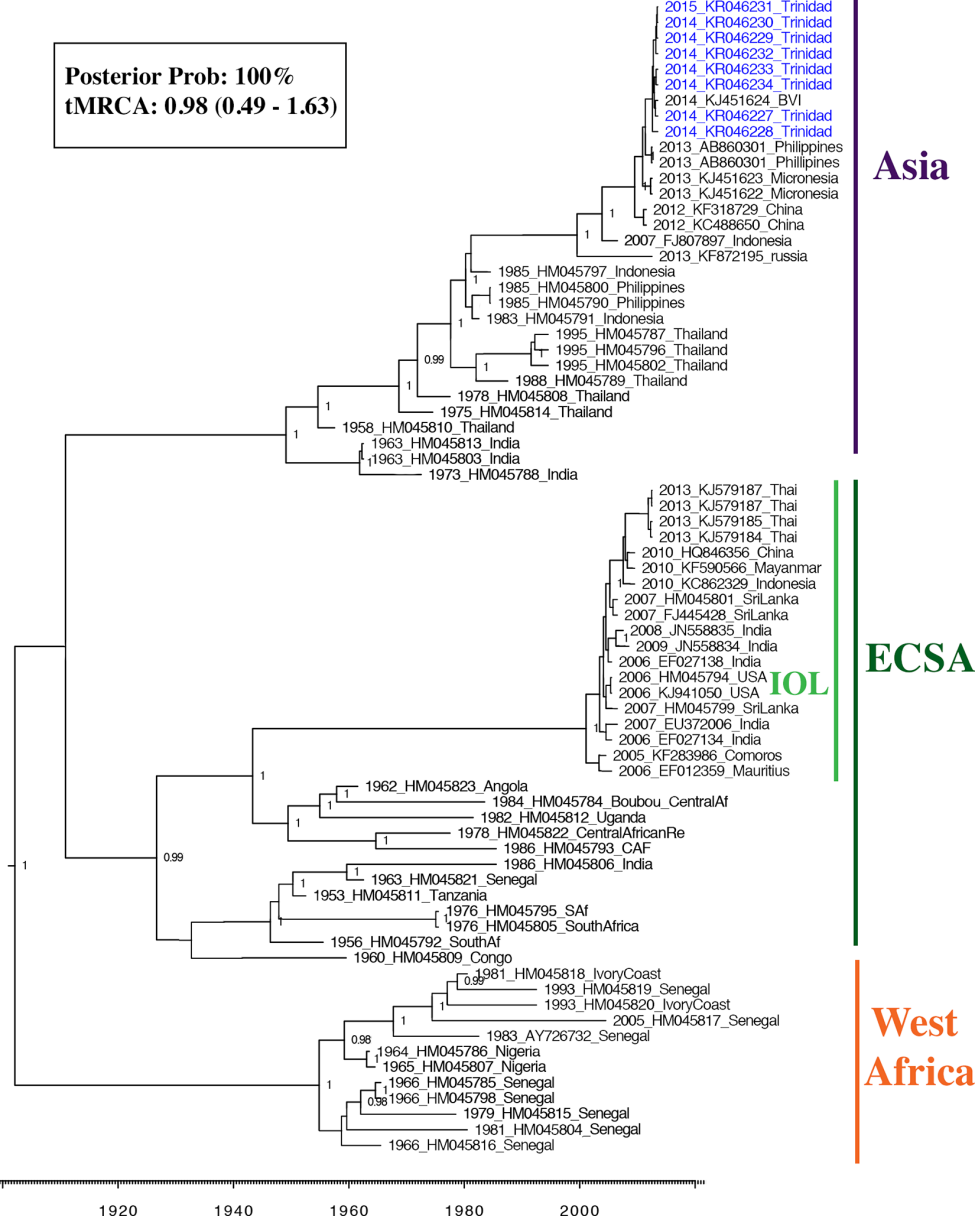
Maximum clade credibility (MCC) phylogeny based on the complete coding region of 74 CHIKV sequences. The three major CHIKV genotypes are labelled, as well as the IOL within the ECSA clade. Sequences generated from this study (Genbank accession numbers (KR046227 –KR046234) are labelled in blue. Nodes with clade credibilities (posterior probabilities) ≥ 0.95 are labelled accordingly. The clade credibility for the node supporting the Trinidad and BVI sequences is shown in the insert together with the mean estimated time to most recent common ancestor (tMRCA) in years (with 95% HPD in brackets).

Using an Illumina platform, complete genome sequences for CHIKV were determined directly from RNA isolated from the sera of 8 individuals. The overall alignment rate of the reads varied for each sample, ranging from 0.45–17.5%. Nucleotide identity amongst the consensus sequences from these individuals was 99.9–100% and 99.8–100% at the amino acid level (S6 Table). Non-synonymous substitutions occurred at nucleotide positions 774 (T→A), 1509 (C→T), 1781 (C→T), 3563 (G→A), 8822 (C→T) and 10,059 (G→A), resulting in changes at aminoacid residues 233 (L→Q), 478 (A→V), 569 (R→C) and 1163 (V→I) in the non-structural polypeptide and at residues 419 (T→I) and 832 (G→R) in the structural polypeptide. The latter two being in the E2 and E1 proteins respectively. Both MCC (Fig 2) and ML phylogenies (S1 Fig) estimated from the data set showed that the Trinidad sequences clustered together with the BVI sequence (Accession no. KJ451624) isolated in 2014 near the beginning of the Caribbean outbreak [39]. The most closely related Asian sequences circulated between 2012 and 2013 in China, the Philippines and Micronesia. The clade containing the Trinidad and BVI sequences was defined by the aforementioned amino acid changes and was estimated to be evolving at a rate of 5 x 10^−3^ substitutions per site per year (95% HPD: 1 x 10^−4–^1 x 10^−3^ substitutions per site per year) with the most recent common ancestor (MRCA) having arisen about 1 year prior to November 2014 `(0.98 years ago [95% higher probability density (HPD): 0.49–1.63]).

For three individuals, sequencing was of sufficient depth to allow reliable reporting of within-host variation at individual nucleotide positions within the non-structural and structural polypeptide genes. Within the non-structural polypeptide ORF, for one individual, at nucleotide position 1228 (i.e. residue 384 of nsp1), 1.1% of reads had A instead of the majority T (p = 2.16 x 10^−7^) both of which encode Leu, and at nucleotide position 3276, 1.6% of reads had T instead of G (p = 1.77 x 10^−13^) which would result in a Ser to Ile substitution at residue 1067 of the nonstructural polypeptide (within nsp2). For the structural polypeptide gene, variation was detected at nucleotide position 9377 (residue 604 i.e. residue 279 in E2) where the minority species was A instead of G. This variant, which was detected in three individuals at frequencies of 2.03, 2.08 and 2.69% (p = 1.48 x 10^−23^, 1.15 x 10^−4^, 5.59 x 10^−5^ respectively), would result in a Gly-to-Glu change. One individual also had a minority variant at nucleotide position 9037 such that 1.18% had T instead of G (p < 6.19 x 10^−2^), which would result in replacement of Glu at amino acid 491 of the structural polypeptide (residue 165 in E2) with a stop codon. None of the four single adaptive mutations in E1 (A226V) and E2 (K252Q; K233E; L210Q) or the synergistic adaptive mutations in E2/E3 (R198Q/S18F) that confer a fitness advantage for IOL strains in *A*. *albopictus* [6,8,40,41] were detected.

## Discussion

Of the 158 individuals enrolled in the study CHIKV was detected by RT-qPCR in 19% (n = 30). Anti-CHIKV IgM antibodies were detected in 6 of the 30 individuals and in 21 individuals who tested negative for CHIKV by RT-qPCR. Therefore 51 individuals (32.3%) had evidence of infection with CHIKV. CHIKV accounted for 7.7%, 4,5%, 36.6%, and 13.3% of cases enrolled in July, August, September and November, respectively. As of August, 2015, 303 laboratory-confirmed cases had been reported in T&T by the Caribbean Public Health Agency [42]. Data on the total number of suspected and probable cases are not available, but it may be presumed to be much higher. Without a reliable estimate of total CHIKV incidence in the country it is difficult to interpret the observed trend in the number of cases detected in this study. The initial increase in number of observed CHIKV cases may be a reflection of the rapid expansion of the virus in Trinidad while the moderate decline in the proportion of CHIKV cases in November compared to September may reflect a decline in incidence or may simply be a result of people becoming less likely to seek hospital care as knowledge about the virus and the nonlethal consequences of infection became more widespread. The Ministry of Health, for example, launched a public education campaign including a “Chikungunya Virus Bus Tour” in August 2014. Because of the overlap with Dengue symptomology the general advice was that individuals showing symptoms should immediately seek healthcare, however most would probably have visited a primary care facility as opposed to hospital emergency care department such as the one where this study was conducted.

Our results confirm that the etiologic CHIKV strain in Trinidad belongs to the Asian genotype and is nearly a direct descendent of the BVI strain isolated in January 2014 [11], with which it shares over 99.9% nucleotide sequence identity across the two ORFs. At the time of writing, no other genomic CHIKV nucleotide sequences from the Americas were available so, although likely, a common origin for CHIKV throughout the Caribbean cannot be confirmed. Additional sequencing would be required to confirm whether other lineages or genotypes have been circulating in the Caribbean as has been the case in Brazil (REF). Analysis of intrahost variation in three individuals indicated the existence of variants with non-synonymous substitutions at low frequency (<3%). None of these corresponded to known adaptive mutations that confer a fitness advantage for IOL strains in *A*. *albopictus* [6,8,40,41].

The data presented arose from a febrile surveillance study for which the initial focus was DENV, however with the emergence of CHIKV in the Americas, specific screening for CHIKV using RT-qPCR was included. Amongst the 158 participants, only 8 were RT-qPCR positive for DENV. However based on the subset of samples (n = 125) that underwent both RT-qPCR and complete ELISA testing, a total of 82 (66%) had evidence of a current or recent DENV infections. Most of the 8 RT-qPCR confirmed DENV infections were DENV-1, which is consistent with the results of PCR-based serotyping of DENV-positive sera derived from the Trinidad Public Health Laboratories from 2011–2014 which showed that DENV-1 and -4 were the dominant serotypes during this period, with DENV-1 being the more common (N. Sahadeo et al., *manuscript in preparation*).

Our findings suggest that persons with confirmed CHIKV infection (i.e. RT-qPCR-positive for CHIKV) visited the APFC roughly one day earlier than those without RT-qPCR evidence of CHIKV. Others have reported significantly earlier presentation of CHIKV infected individuals at health care institutions compared to DENV infected individuals [43]. This may be a consequence of the more severe joint pain associated with CHIKV infection, which in the current study was more commonly reported in individuals with confirmed CHIKV infection than in individuals negative for CHIKV and DENV by RT-qPCR, although in the case of DENV the difference was not significant.

Several studies have reported leukocytosis as more likely to be present in chikungunya cases, and leucopenia and thrombocytopenia being more likely to be present in dengue cases [43–50]. In particular, thrombocytopenia is considered a hallmark of DENV infection [51]. In the current study, bivariable analysis indicated lower mean white blood cell counts in individuals with confirmed CHIKV infection compared to individuals with AUFI that were negative for CHIKV by RT-qPCR. However the extent of leucopenia in the those confirmed CHIKV cases was less than observed in the 88 individuals with confirmed DENV infection (i.e. positive for DENV by RT-qPCR), who had significantly lower white blood cell and platelet counts than individuals with confirmed CHIKV infection.

There have been concerns that the arrival of CHIKV would result in life-threatening DENV cases being overlooked, as the two are clinically indistinguishable during the early stages. This concern was critical because timely confirmatory laboratory testing is largely unavailable and rapid “point-of-care” approaches can be costly and unreliable. Our data suggest that platelet counts, which are routinely performed at the EWMSC and other major healthcare institutions in T&T, are useful for identifying individuals who are more likely to have DENV than CHIKV. Our data indicate that in addition to being lower than in individuals with confirmed CHIKV infection, the mean platelet count in the 8 individuals with confirmed DENV infection was at a level (<150 x 10^3^/μl) at which the EWMSC would normally recommend further monitored. Additionally, out of those 8 individuals, five had platelet counts below this threshold.

Furthermore, we investigated the feasibility of using combinations of clinical features as a means of differentiating between individuals with confirmed and probable CHIKF (i.e. with evidence of CHIKV infection by PCR and / or ELISA) and individuals with confirmed and recent probable DF. The combination rash, joint pain and white blood cell count <7 x10^3^/μl was most efficient with a PPV and NPV of 77% and 96% respectively and, a sensitivity and specificity of 94% and 84% respectively. Rash, joint pain and white blood cell count <7 x10^3^/μl together were also most efficient at differentiating CHIKF from other AUFIs. In this case the combination had a PPV and NPV of 58% and 95% respectively and, a sensitivity and specificity of 88% and 78% respectively. Considering the mean PLT count was lower (<150 x 10^3^/μl) in individuals positive for DENV by RT-qPCR, a profile using these 4 clinical features might be suitable as a means of differentiating between those with CHIKV infection and those with DENV infection.

### Strengths and limitations

In this study participants were solicited three days per week from amongst individuals identified by attending physicians as meeting the criteria of having been febrile within the last seven days. We cannot rule out selection bias at this interface and so cannot generalise our findings to those who were not interviewed. However as it was a study of AUFI and was not restricted to persons with a specific infection, selection bias among those who thought they had DENV or CHIKV may be less of a concern. Three interviewers were involved in the administration of questionnaires to participants of the study. In order to ensure consistency, they were trained in the delivery of questions and recording of responses but the possibility of interviewer bias cannot be completely excluded.

This study was performed at a tertiary level public healthcare facility, and the distribution of risk factors may be different among those who sought care at public primary care facilities (i.e. local health centres) or at other private healthcare facilities. Importantly, despite having a small sample size for the comparison of persons with and without CHIKV, there was sufficient power to detect several meaningful associations on bivariable and multivariable analysis. For the sub-analysis comparing the symptoms of those with CHIKV or DENV, the sample size was restricted to 38 and thus analyses were underpowered. In spite of this, there appeared to be differences in the symptomatic presentation of persons infected with either.

### Conclusion

In summary, our study confirms the presence of Asian genotype CHIKV in Trinidad and identifies symptoms that distinguish individuals with acute fevers who are viraemic for CHIKV from those who are CHIKV negative by RT-qPCR, and identifies laboratory and clinical features that distinguish between those who are viraemic for CHIKV versus DENV. 51 (32.3%) individuals had evidence of CHIKV infection (positive for CHIKV by RT-qPCR and / or positive for anti-CHIKV IgM antibodies by ELISA). Of these, only 30 were positive for CHIKV by RT-qPCR. While all individuals reported being within 7 days of onset of their symptoms (when viraemia is often detectable) it is possible that there were individuals in whom viraemia was already cleared or too low to be detected. Additionally, future studies aimed at determining population-level seroprevalence will be essential in order to get an accurate estimate of the impact (economic and otherwise) of CHIKV on the Trinidad population and to predict the potential impact of future outbreaks. It will also be important to monitor the genotypes in circulation given the potential for accumulation of adaptive mutations demonstrated by some strains [6,8,40,41].

The rapid spread of the newly emerged CHIKV in the Caribbean in 2014 and the resulting epidemic demonstrate the dramatic effect of the introduction of a “novel” virus on a naïve population, especially where vectors are endemic. Public health authorities in the Americas are, as of August 2015, on alert for the emergence of Zika virus, a flavivirus known to cause widespread outbreaks of dengue-like illness in Africa and Asia, after being recently detected in the region for the first time. As commercial air travel expands this will continue to facilitate the movement of viruses and ongoing monitoring of individuals with AUFIs is essential to rapidly detect the emergence of viruses to non-endemic areas.

## Supporting Information

S1 Fig Maximum Likelihood phylogeny of CHIKV complete coding region of 74 CHIKV sequences. The three major CHIKV genotypes are labelled, as well as the IOL within the ECSA clade. Sequences generated from this study (Genbank accession numbers KR046227 –KR046234) are labelled in in grey. Nodes supported by bootstrap values ≥ 0.95 are labelled accordingly.
(TIF)

S1 Table Sequences included in data set for phylogenetic analysis. (DOCX)

S2 Table Frequencies of characteristics of the patients of the Adult Primary Care Facility of the Eric Williams Medical Sciences Complex, Trinidad & Tobago (Dec 2013–Nov 2014). (DOCX)

S3 Table DENV status based on DENV specific IgM ELISA, IgG ELISA and RT-qPCR for 125 individuals with sufficient testing. (DOCX)

S4 Table Bivariable associations of patients of the Adult Primary Care Facility of the Eric Williams Medical Sciences Complex, Trinidad & Tobago, (Dec 2013–Nov 2014) with having a confirmed infection^ⱡ^ with Chikungunya virus (CHIKV). (DOCX)

S5 Table Bivariable associations of patients of the Adult Primary Care Facility of the Eric Williams Medical Sciences Complex, Trinidad & Tobago, (Dec 2013–Nov 2014) with having a confirmed infection^ⱡ^ with either any Dengue virus serotype (DENV) or Chikungunya virus (CHIKV). (DOCX)

S6 Table Percentage nucleotide and amino acid identities across the open reading frame of CHIKV sequences from Trinidad and the British Virgin Islands. (DOCX)
