# Correction: Molecular Characterisation of Chikungunya Virus Infections in Trinidad and Comparison of Clinical and Laboratory Features with Dengue and Other Acute Febrile Cases

**DOI:** 10.1371/journal.pntd.0004305

**Published:** 2015-12-11

**Authors:** Nikita Sahadeo, Hamish Mohammed, Orchid M. Allicock, Albert J. Auguste, Steven G. Widen, Kimberly Badal, Krishna Pulchan, Jerome E. Foster, Scott C. Weaver, Christine V. F. Carrington

There is an error in the third sentence of the second paragraph of the Discussion section. At the end of this sentence there should be a link to Reference 17 instead of reading (REF). The correct sentence is: Additional sequencing would be required to confirm whether other lineages or genotypes have been circulating in the Caribbean as has been the case in Brazil [17].
