# Supplementary material for: Molecular Characterisation of Chikungunya Virus Infections in Trinidad and Comparison of Clinical and Laboratory Features with Dengue and Other Acute Febrile Cases
Source: PLoS Negl Trop Dis. 2015 Nov 18;9(11):e0004199. doi: 10.1371/journal.pntd.0004199 (PMC4651505; doi:10.1371/journal.pntd.0004199)
Supplement: S1 Table — (DOCX) [file pntd.0004199.s002.docx]

Supplementary Table 1 - Sequences included in data set for phylogenetic analysis

| Year | Accession Number | Country |
| --- | --- | --- |
| 1953 | HM045811 | Tanzania |
| 1956 | HM045792 | South Africa |
| 1958 | HM045810 | Thailand |
| 1960 | HM045809 | Congo |
| 1962 | HM045823 | Angola |
| 1963 | HM045813 | India |
| 1963 | HM045803 | India |
| 1963 | HM045821 | Senegal |
| 1964 | HM045786 | Nigeria |
| 1965 | HM045807 | Nigeria |
| 1966 | HM045798 | Senegal |
| 1966 | HM045816 | Senegal |
| 1966 | HM045785 | Senegal |
| 1973 | HM045788 | India |
| 1975 | HM045814 | Thailand |
| 1976 | HM045795 | South Africa |
| 1976 | HM045805 | South Africa |
| 1978 | HM045822 | Central African Region |
| 1978 | HM045808 | Thailand |
| 1979 | HM045815 | Senegal |
| 1981 | HM045818 | Ivory Coast |
| 1981 | HM045804 | Senegal |
| 1982 | HM045812 | Uganda |
| 1983 | HM045791 | Indonesia |
| 1983 | AY726732 | Senegal |
| 1984 | HM045784 | Central African Region |
| 1985 | HM045797 | Indonesia |
| 1985 | HM045790 | Philippines |
| 1985 | HM045800 | Philippines |
| 1986 | HM045793 | Central African Region |
| 1986 | HM045806 | India |
| 1986 | HM045793 | Central African Region |
| 1988 | HM045789 | Thailand |
| 1993 | HM045820 | Ivory Coast |
| 1993 | HM045819 | Senegal |
| 1995 | HM045802 | Thailand |
| 1995 | HM045796 | Thailand |
| 1995 | HM045787 | Thailand |
| 2005 | KF283986 | Comoros |
| 2005 | HM045817 | Senegal |
| 2006 | EF027134 | India |
| 2006 | EF027138 | India |
| 2006 | EF012359 | Mauritius |
| 2006 | KJ941050 | USA |
| 2006 | HM045794 | USA |
| 2007 | EU372006 | India |
| 2007 | FJ807897 | Indonesia |
| 2007 | FJ445428 | Sri Lanka |
| 2007 | HM045801 | Sri Lanka |
| 2007 | HM045799 | Sri Lanka |
| 2008 | JN558835 | India |
| 2009 | JN558834 | India |
| 2010 | HQ846356 | China |
| 2010 | KC862329 | Indonesia |
| 2010 | KF590566 | Myanmar |
| 2012 | KF318729 | China |
| 2012 | KC488650 | China |
| 2013 | KJ451622 | Micronesia |
| 2013 | KJ451623 | Micronesia |
| 2013 | AB860301 | Philippines |
| 2013 | AB860301 | Philippines |
| 2013 | KF872195 | Russia |
| 2013 | KJ579187 | Thailand |
| 2013 | KJ579185 | Thailand |
| 2013 | KJ579184 | Thailand |
| 2013 | KJ579187 | Thailand |
| 2014 | VE57_2 | Trinidad and Tobago |
| 2014 | VE55_4 | Trinidad and Tobago |
| 2014 | VE56_9 | Trinidad and Tobago |
| 2014 | VE56_13 | Trinidad and Tobago |
| 2014 | VE54_19 | Trinidad and Tobago |
| 2014 | VE53_20 | Trinidad and Tobago |
| 2014 | VE56_20 | Trinidad and Tobago |
| 2014 | VE54_20 | Trinidad and Tobago |
| 2014 | KJ451624 | British Virgin Islands |
