# Supplementary material for: Molecular Characterisation of Chikungunya Virus Infections in Trinidad and Comparison of Clinical and Laboratory Features with Dengue and Other Acute Febrile Cases
Source: PLoS Negl Trop Dis. 2015 Nov 18;9(11):e0004199. doi: 10.1371/journal.pntd.0004199 (PMC4651505; doi:10.1371/journal.pntd.0004199)
Supplement: S2 Table — (DOCX) [file pntd.0004199.s003.docx]

Supplementary Table 2 - Frequencies of characteristics of the patients of the Adult Primary Care Facility of the Eric Williams Medical Sciences Complex, Trinidad & Tobago (Dec 2013–Nov 2014)

|  | n | % |
| --- | --- | --- |
| *Demographics* |  |  |
| Age, years (categorised on median) |  |  |
| <32 | 75 | 47.5 |
| >32 | 83 | 52.5 |
| Sex |  |  |
| Male | 79 | 50 |
| Female | 79 | 50 |
| Ethnicity |  |  |
| Afro-Trinidadian | 38 | 24.1 |
| Indo-Trinidadian | 35 | 22.2 |
| Mixed | 30 | 19 |
| Other | 11 | 7 |
| Missing | 44 | 27.8 |
| Marital status |  |  |
| Not married^a^ | 65 | 41.1 |
| Married (including common-law) | 83 | 52.5 |
| Missing | 10 | 6.3 |
| Education level |  |  |
| >Secondary school | 47 | 29.7 |
| <Secondary school | 89 | 56.3 |
| Missing | 22 | 13.9 |
| Employment status |  |  |
| Unemployed | 37 | 23.4 |
| Employed | 109 | 69 |
| Missing | 12 | 7.6 |
| Income stability |  |  |
| Not daily paid | 100 | 63.3 |
| Daily paid | 3 | 1.9 |
| Missing | 55 | 34.8 |
| Nationality |  |  |
| Not Trinidadian or Tobagonian | 34 | 21.5 |
| Trinidadian or Tobagonian | 124 | 78.5 |
| *Risk factors for viral infection* |  |  |
| Travelled outside Trinidad in the 2 weeks prior to interview |  |  |
| No | 139 | 88 |
| Yes | 11 | 7 |
| Missing | 8 | 5.1 |
| Visited a forested area in Trinidad or Tobago in the 2 weeks prior to interview |  |  |
| No | 98 | 62 |
| Yes | 50 | 31.6 |
| Missing | 10 | 6.3 |
| Contact with a wild animal in the 2 weeks prior to interview |  |  |
| No | 149 | 94.3 |
| Yes | 9 | 5.7 |
| Contact with livestock in the 2 weeks prior to interview |  |  |
| No | 140 | 88.6 |
| Yes | 17 | 10.8 |
| Missing | 1 | 0.6 |
| Contact with a companion animal in the 2 weeks prior to interview |  |  |
| No | 99 | 62.7 |
| Yes | 58 | 36.7 |
| Missing | 1 | 0.6 |
| Previous dengue |  |  |
| No | 118 | 74.7 |
| Yes | 34 | 21.5 |
| Missing | 6 | 3.8 |
| Dengue previously confirmed by laboratory test |  |  |
| Yes | 33 | 20.9 |
| Missing | 125 | 79.1 |
| Febrile household member in the 2 weeks prior to interview |  |  |
| No | 104 | 65.8 |
| Yes | 40 | 25.3 |
| Missing | 14 | 8.9 |
| Household member diagnosed with dengue in the 2 weeks prior to interview |  |  |
| No | 131 | 82.9 |
| Yes | 17 | 10.8 |
| Missing | 10 | 6.3 |
| History of mosquito bites at home |  |  |
| No | 29 | 18.4 |
| Yes | 119 | 75.3 |
| Missing | 10 | 6.3 |
| Screened windows at home |  |  |
| No | 126 | 79.7 |
| Yes | 21 | 13.3 |
| Missing | 11 | 7 |
| Storage of water at home |  |  |
| No | 63 | 39.9 |
| Yes | 87 | 55.1 |
| Missing | 8 | 5.1 |
| Bushy or unkempt areas around home |  |  |
| No | 78 | 49.4 |
| Yes | 72 | 45.6 |
| Missing | 8 | 5.1 |
| Coworker diagnosed with dengue in the 2 weeks prior to interview |  |  |
| No | 99 | 62.7 |
| Yes | 20 | 12.7 |
| Missing | 39 | 24.7 |
| *Clinical factors* |  |  |
| History of yellow fever virus vaccination |  |  |
| No | 95 | 60.1 |
| Yes | 63 | 39.9 |
| History of Meales-Mumps-Rubella vaccination |  |  |
| No | 109 | 69 |
| Yes | 49 | 31 |
| History of Hepatitis B vaccination |  |  |
| No | 122 | 77.2 |
| Yes | 36 | 22.8 |
| History of other vaccination |  |  |
| No | 120 | 75.9 |
| Yes | 38 | 24.1 |
| History of bleeding disorder |  |  |
| No | 137 | 86.7 |
| Yes | 6 | 3.8 |
| Missing | 15 | 9.5 |
| Admitted to hospital |  |  |
| No | 15 | 9.5 |
| Yes | 18 | 11.4 |
| Missing | 125 | 79.1 |
| Headache |  |  |
| No | 27 | 17.1 |
| Yes | 131 | 82.9 |
| Muscle pain |  |  |
| No | 56 | 35.4 |
| Yes | 102 | 64.6 |
| Joint pain |  |  |
| No | 55 | 34.8 |
| Yes | 103 | 65.2 |
| Back pain |  |  |
| No | 149 | 94.3 |
| Yes | 9 | 5.7 |
| Rash |  |  |
| No | 124 | 78.5 |
| Yes | 34 | 21.5 |
| Fatigue |  |  |
| No | 94 | 59.5 |
| Yes | 64 | 40.5 |
| Eye pain |  |  |
| No | 80 | 50.6 |
| Yes | 78 | 49.4 |
| Cough |  |  |
| No | 114 | 72.2 |
| Yes | 44 | 27.8 |
| Nausea |  |  |
| No | 117 | 74.1 |
| Yes | 41 | 25.9 |
| Vomiting |  |  |
| No | 113 | 71.5 |
| Yes | 45 | 28.5 |
| Diarrhoea |  |  |
| No | 120 | 75.9 |
| Yes | 38 | 24.1 |
| Sore throat |  |  |
| No | 128 | 81 |
| Yes | 30 | 19 |
| Weakness |  |  |
| No | 52 | 32.9 |
| Yes | 106 | 67.1 |
| Stiff neck |  |  |
| No | 137 | 86.7 |
| Yes | 21 | 13.3 |
| Dizziness |  |  |
| No | 108 | 68.4 |
| Yes | 50 | 31.6 |
| Disorientation |  |  |
| No | 135 | 85.4 |
| Yes | 23 | 14.6 |
| Abdominal pain |  |  |
| No | 116 | 73.4 |
| Yes | 42 | 26.6 |
| Nose bleed |  |  |
| No | 155 | 98.1 |
| Yes | 3 | 1.9 |
| Gum bleed |  |  |
| No | 152 | 96.2 |
| Yes | 6 | 3.8 |
| Abnormal vaginal bleeding |  |  |
| No | 150 | 94.9 |
| Yes | 8 | 5.1 |
| Blood in urine |  |  |
| No | 152 | 96.2 |
| Yes | 6 | 3.8 |
| Bruising |  |  |
| No | 156 | 98.7 |
| Yes | 2 | 1.3 |
| Blood in stool |  |  |
| No | 155 | 98.1 |
| Yes | 3 | 1.9 |
| Any haemorrhagic manifestation^b^ |  |  |
| No | 132 | 83.5 |
| Yes | 26 | 16.5 |
| *Laboratory results* |  |  |
| Dengue virus (DENV) – Immunoglobulin (Ig) M Enzyme Linked Immunofluorescent Assay (ELISA) |  |  |
| No | 77 | 48.7 |
| Yes | 48 | 30.4 |
| Missing | 33 | 20.9 |
| DENV – IgM ELISA (Accident & Emergency Department, A&E) |  |  |
| No | 38 | 24.1 |
| Yes | 45 | 28.5 |
| Missing | 75 | 47.5 |
| DENV – IgG ELISA |  |  |
| No | 68 | 43 |
| Yes | 57 | 36.1 |
| Missing | 33 | 20.9 |
| DENV – IgG ELISA (A&E) |  |  |
| No | 28 | 17.7 |
| Yes | 55 | 34.8 |
| Missing | 75 | 47.5 |
| DENV – Real Time Polymerase Chain Reaction (RT PCR) |  |  |
| No | 150 | 94.9 |
| Yes | 8 | 5.1 |
| Missing |  |  |
| Chikungunya virus (CHIKV) – IgM ELISA (A&E) |  |  |
| No | 28 | 17.7 |
| Yes | 1 | 0.6 |
| Missing | 129 | 81.6 |
| CHIKV – RT PCR |  |  |
| No | 128 | 81 |
| Yes | 30 | 19 |
|  | Mean | Min-Max^‡^ |
| Days post onset of illness | 3.5 | 0.0-7.0 |
| Days post onset of fever | 3.16 | 0.0-7.0 |
| Temperature (^°^C) | 37.8 | 36.0-40.0 |
| White blood cell count (10^3^/μl) | 7.99 | 1.6-28.0 |
| Haematocrit (%) | 42.83 | 12.9-284.0 |
| Platelet count (10^3^/μl) | 217.6 | 16.0-712.0 |

^‡^ Minimum - Maximum

^a^ Includes: single, divorced, and widower

^b^ Includes: nose bleed, gum bleed, abnormal vaginal bleeding, blood in urine, bruising or blood in stool.
