# Supplementary material for: Molecular Characterisation of Chikungunya Virus Infections in Trinidad and Comparison of Clinical and Laboratory Features with Dengue and Other Acute Febrile Cases
Source: PLoS Negl Trop Dis. 2015 Nov 18;9(11):e0004199. doi: 10.1371/journal.pntd.0004199 (PMC4651505; doi:10.1371/journal.pntd.0004199)
Supplement: S3 Table — (DOCX) [file pntd.0004199.s004.docx]

Supplementary Table 3 - DENV status based on DENV specific IgM ELISA, IgG ELISA and RT-qPCR for 125 individuals with sufficient testing.

| Sample | IGM  ELISA  Result | IgG  ELISA  Result | DENV  RT-PCR Result | DENV status | Sample | IGM  ELISA  Result | IgG  ELISA  Result | DENV  RT-PCR  Result | DENV status |
| --- | --- | --- | --- | --- | --- | --- | --- | --- | --- |
| EW-160914-06 | N | N | N | DENV neg | EW-150914-06 | N | P | N |  |
| EW-041114-02 | N | P | N | Recent probable | EW-150914-07 | N | P | N |  |
| EW-170914-DM | N | E | N |  | EW-160814-02 | N | P | N |  |
| EW-160914-03 | N | N | N |  | EW-300714-01 | N | P | N |  |
| EW-240914-01 | N | P | N |  | EW-030114-04 | N | N | N |  |
| EW-240914-02 | N | N | N |  | EW-190914-04 | N | P | N |  |
| EW-190914-01 | N | N | N |  | EW-160914-02 | N | P | N |  |
| EW-180914-04 | N | P | N |  | EW-160914-04 | N | E | N |  |
| EW-260714-01 | N | N | N |  | EW-160914-07 | N | N | N |  |
| EW-301213-02 | N | N | N |  | EW-170714-SR | N | P | N |  |
| EW-180914-03 | N | P | N |  | EW-100514-01 | N | N | N |  |
| EW-180914-05 | N | N | N |  | EW-301213-03 | N | N | N |  |
| EW-150914-04 | N | P | N |  | EW-051114-01 | N | P | N |  |
| EW-040414-02 | N | N | N |  | EW-120914-04 | N | N | N |  |
| EW-220414-02 | N | N | N |  | EW-300814-02 | N | N | N |  |
| EW-041114-03 | N | P | N |  | EW-160814-01 | N | N | N |  |
| EW-250914-03 | N | P | N |  | EW-130814-01 | N | N | N |  |
| EW-230914-02 | N | P | N |  | EW-060114-03 | N | N | N |  |
| EW-200914-02 | N | E | N |  | EW-150914-02 | N | N | N |  |
| EW-160914-01 | N | P | N |  | EW-080914-03 | N | P | N |  |
| EW-250814-01 | N | N | N |  | EW-080914-04 | N | N | N |  |
| EW-070214-02 | N | P | N |  | EW-200714-MW | N | N | N |  |
| EW-200914-04 | N | P | N |  | EW-170714-JP | N | N | N |  |
| EW-190914-02 | N | P | N |  | EW-140714-01 | N | P | N |  |
| EW-190914-03 | N | P | N |  | EW-160814-03 | N | P | N |  |
| EW-180914-01 | N | N | N |  | EW-120914-03 | N | N | N |  |
| EW-180914-02 | N | N | N |  | EW-270814-03 | N | N | N |  |
| EW-270814-02 | N | N | N |  | EW-050814-04 | N | P | N |  |
| EW-120814-02 | N | P | N |  | EW-100214-01 | N | N | N |  |
| EW-041114-01 | N | E | N |  | EW-070214-01 | N | N | N |  |
| EW-031114-03 | N | P | N |  | EW-230114-01 | N | N | N |  |
| EW-300714-02 | N | P | N |  | EW-080914-02 | N | N | N |  |
| EW-260714-02 | N | P | N |  | EW-040914-03 | N | P | N |  |
| EW-100714-01 | N | N | N |  | EW-040914-04 | N | N | N |  |
| EW-050414-01 | N | P | N |  | EW-170114-04 | N | P | N |  |
| EW-200914-10 | N | N | N |  | EW-030114-02 | N | P | N |  |
| EW-030914-01 | N | N | N |  | EW-020914-03 | N | N | N |  |
| EW-120814-01 | N | P | N |  | EW-100914-03 | N | N | N |  |
| EW-250914-02 | P | P | N |  | EW-170914-DBM | P | P | N |  |
| EW-160914-08 | P | N | N |  | EW-200914-05 | P | P | N |  |
| EW-200914-11 | P | P | N |  | EW-220414-05 | P | N | N |  |
| EW-250914-01 | P | P | N |  | EW-040514-02 | P | N | N |  |
| EW-230914-03 | P | N | N |  | EW-300414-01 | P | N | N |  |
| EW-301213-01 | P | N | N |  | EW-120814-03 | P | N | N |  |
| EW-160814-04 | P | N | N |  | EW-190514-01 | P | N | N |  |
| EW-030114-03 | P | N | P |  | EW-160914-05 | P | P | N |  |
| EW-300414-02 | P | P | N |  | EW-080914-01 | P | P | N |  |
| EW-270114-01 | P | N | N |  | EW-200114-01 | P | N | N |  |
| EW-070114-02 | P | P | N |  | EW-030114-01 | P | N | P |  |
| EW-150914-03 | P | P | N |  | EW-050814-01 | P | N | N |  |
| EW-050814-02 | P | N | N |  | EW-050814-03 | P | P | N |  |
| EW-060414-04 | P | N | P |  | EW-090914-01 | P | P | N |  |
| EW-160814-05 | P | N | N |  | EW-220714-01 | P | N | N |  |
| EW-140214-01 | P | P | N |  | EW-270114-02 | P | P | P |  |
| EW-170114-01 | P | N | N |  | EW-170114-02 | P | N | N |  |
| EW-070814-01 | P | N | N |  | EW-100214-03 | P | N | N |  |
| EW-310114-02 | P | P | P |  | EW-070114-01 | P | P | P |  |
| EW-200114-02 | P | N | N |  | EW-301213-04 | P | N | N |  |
| EW-100914-01 | P | N | N |  | EW-040914-02 | P | P | N |  |
| EW-180314-02 | P | P | N |  | EW-100314-02 | P | P | N |  |
| EW-100114-01 | P | P | N |  | EW-040914-01 | P | P | N |  |
| EW-030214-02 | P | P | N |  |  |  |  |  |  |
