# Supplementary material for: Molecular Characterisation of Chikungunya Virus Infections in Trinidad and Comparison of Clinical and Laboratory Features with Dengue and Other Acute Febrile Cases
Source: PLoS Negl Trop Dis. 2015 Nov 18;9(11):e0004199. doi: 10.1371/journal.pntd.0004199 (PMC4651505; doi:10.1371/journal.pntd.0004199)
Supplement: S4 Table — (DOCX) [file pntd.0004199.s005.docx]

Supplementary Table 4 - Bivariable associations of patients of the Adult Primary Care Facility of the Eric Williams Medical Sciences Complex, Trinidad & Tobago, (Dec 2013–Nov 2014) with having a confirmed infection^ⱡ^ with Chikungunya virus (CHIKV)

|  | CHIKV Not confirmed | | CHIKV Confirmed | |  |
| --- | --- | --- | --- | --- | --- |
|  | n | (%) | n | (%) | *p*-value |
| Demographics |  |  |  |  |  |
| Age, years (categorised on median) |  |  |  |  |  |
| <32 | 64 | (50.0) | 11 | (36.7) | 0.188 |
| >32 | 64 | (50.0) | 19 | (63.3) |  |
| Sex |  |  |  |  |  |
| Male | 68 | (53.1) | 11 | (36.7) | 0.105 |
| Female | 60 | (46.9) | 19 | (63.3) |  |
| Ethnicity |  |  |  |  |  |
| Afro-Trinidadian | 30 | (32.6) | 8 | (36.4) | 0.419 |
| Indo-Trinidadian | 30 | (32.6) | 5 | (22.7) |  |
| Mixed | 25 | (27.2) | 5 | (22.7) |  |
| Other | 7 | (7.6) | 4 | (18.4) |  |
| Marital status |  |  |  |  |  |
| Not married^a^ | 58 | (47.5) | 7 | (26.9) | 0.054 |
| Married (including common-law) | 64 | (52.5) | 19 | (73.1) |  |
| Education level |  |  |  |  |  |
| >Secondary school | 40 | (35.7) | 7 | (29.2) | 0.540 |
| <Secondary school | 72 | (64.3) | 17 | (70.8) |  |
| Employment status |  |  |  |  |  |
| Unemployed | 27 | (22.9) | 10 | (35.7) | 0.160 |
| Employed | 91 | (77.1) | 18 | (64.3) |  |
| Income stability |  |  |  |  |  |
| Not daily paid | 83 | (96.5) | 17 | (100.0) | 1.000 |
| Daily paid | 3 | (3.5) | 0 | (0.0) |  |
| Nationality |  |  |  |  |  |
| Not Trinidadian or Tobagonian | 24 | (18.8) | 10 | (33.3) | 0.080 |
| Trinidadian or Tobagonian | 104 | (81.3) | 20 | (66.7) |  |
| Risk factors |  |  |  |  |  |
| Travelled outside of Trinidad in the 2 weeks prior to interview |  |  |  |  |  |
| No | 114 | (95.0) | 25 | (83.3) | 0.044* |
| Yes | 6 | (5.0) | 5 | (16.7) |  |
| Visited a forested area in Trinidad or Tobago in the 2 weeks prior to interview |  |  |  |  |  |
| No | 72 | (60.5) | 26 | (89.7) | 0.003** |
| Yes | 47 | (39.5) | 3 | (10.3) |  |
| Contact with a wild animal in the 2 weeks prior to interview |  |  |  |  |  |
| No | 120 | (93.8) | 29 | (96.7) | 1.000 |
| Yes | 8 | (6.3) | 1 | (3.3) |  |
| Contact with livestock in the 2 weeks prior to interview |  |  |  |  |  |
| No | 112 | (88.2) | 28 | (93.3) | 0.531 |
| Yes | 15 | (11.8) | 2 | (6.7) |  |
| Contact with a companion animal in the 2 weeks prior to interview |  |  |  |  |  |
| No | 79 | (62.2) | 20 | (66.7) | 0.649 |
| Yes | 48 | (37.8) | 10 | (33.3) |  |
| Previous dengue |  |  |  |  |  |
| No | 91 | (74.0) | 27 | (93.1) | 0.026* |
| Yes | 32 | (26.0) | 2 | (6.9) |  |
| Febrile household member in the 2 weeks prior to interview |  |  |  |  |  |
| No | 84 | (72.4) | 20 | (71.4) | 0.917 |
| Yes | 32 | (27.6) | 8 | (28.6) |  |
| Household member diagnosed with dengue in the 2 weeks prior to interview |  |  |  |  |  |
| No | 105 | (88.2) | 26 | (89.7) | 1.000 |
| Yes | 14 | (11.8) | 3 | (10.3) |  |
| History of mosquito bites at home |  |  |  |  |  |
| No | 24 | (20.3) | 5 | (16.7) | 0.651 |
| Yes | 94 | (79.7) | 25 | (83.3) |  |
| Screened windows at home |  |  |  |  |  |
| No | 101 | (86.3) | 25 | (83.3) | 0.770 |
| Yes | 16 | (13.7) | 5 | (16.7) |  |
| Storage of water at home |  |  |  |  |  |
| No | 53 | (44.2) | 10 | (33.3) | 0.282 |
| Yes | 67 | (55.8) | 20 | (66.7) |  |
| Bushy or unkempt areas around home |  |  |  |  |  |
| No | 63 | (52.5) | 15 | (50.0) | 0.806 |
| Yes | 57 | (47.5) | 15 | (50.0) |  |
| Coworker diagnosed with dengue in the 2 weeks prior to interview |  |  |  |  |  |
| No | 82 | (83.7) | 17 | (81.0) | 0.752 |
| Yes | 16 | (16.3) | 4 | (19.0) |  |
| Clinical factors |  |  |  |  |  |
| History of yellow fever virus vaccination |  |  |  |  |  |
| No | 74 | (57.8) | 21 | (70.0) | 0.220 |
| Yes | 54 | (42.2) | 9 | (30.0) |  |
| History of Measles-Mumps-Rubella vaccination |  |  |  |  |  |
| No | 87 | (68.0) | 22 | (73.3) | 0.567 |
| Yes | 41 | (32.0) | 8 | (26.7) |  |
| History of Hepatitis B vaccination |  |  |  |  |  |
| No | 99 | (77.3) | 23 | (76.7) | 0.937 |
| Yes | 29 | (22.7) | 7 | (23.3) |  |
| History of other vaccination |  |  |  |  |  |
| No | 95 | (74.2) | 25 | (83.3) | 0.293 |
| Yes | 33 | (25.8) | 5 | (16.7) |  |
| History of bleeding disorder |  |  |  |  |  |
| No | 109 | (95.6) | 28 | (96.6) | 1.000 |
| Yes | 5 | (4.4) | 1 | (3.4) |  |
| Admitted to hospital |  |  |  |  |  |
| No | 14 | (45.2) | 1 | (50.0) | 1.000 |
| Yes | 17 | (54.8) | 1 | (50.0) |  |
| Headache |  |  |  |  |  |
| No | 20 | (15.6) | 7 | (23.3) | 0.313 |
| Yes | 108 | (84.4) | 23 | (76.7) |  |
| Muscle pain |  |  |  |  |  |
| No | 47 | (36.7) | 9 | (30.0) | 0.489 |
| Yes | 81 | (63.3) | 21 | (70.0) |  |
| Joint pain |  |  |  |  |  |
| No | 50 | (39.1) | 5 | (16.7) | 0.020* |
| Yes | 78 | (60.9) | 25 | (83.3) |  |
| Back pain |  |  |  |  |  |
| No | 119 | (93.0) | 30 | (100.0) | 0.209 |
| Yes | 9 | (7.0) | 0 | (0.0) |  |
| Rash |  |  |  |  |  |
| No | 104 | (81.3) | 20 | (66.7) | 0.080 |
| Yes | 24 | (18.8) | 10 | (33.3) |  |
| Fatigue |  |  |  |  |  |
| No | 79 | (61.7) | 15 | (50.0) | 0.239 |
| Yes | 49 | (38.3) | 15 | (50.0) |  |
| Eye pain |  |  |  |  |  |
| No | 62 | (48.4) | 18 | (60.0) | 0.254 |
| Yes | 66 | (51.6) | 12 | (40.0) |  |
| Cough |  |  |  |  |  |
| No | 91 | (71.1) | 23 | (76.7) | 0.540 |
| Yes | 37 | (28.9) | 7 | (23.3) |  |
| Nausea |  |  |  |  |  |
| No | 93 | (72.7) | 24 | (80.0) | 0.409 |
| Yes | 35 | (27.3) | 6 | (20.0) |  |
| Vomiting |  |  |  |  |  |
| No | 91 | (71.1) | 22 | (73.3) | 0.807 |
| Yes | 37 | (28.9) | 8 | (26.7) |  |
| Diarrhoea |  |  |  |  |  |
| No | 95 | (74.2) | 25 | (83.3) | 0.293 |
| Yes | 33 | (25.8) | 5 | (16.7) |  |
| Sore throat |  |  |  |  |  |
| No | 99 | (77.3) | 29 | (96.7) | 0.015* |
| Yes | 29 | (22.7) | 1 | (3.3) |  |
| Weakness |  |  |  |  |  |
| No | 43 | (33.6) | 9 | (30.0) | 0.706 |
| Yes | 85 | (66.4) | 21 | (70.0) |  |
| Stiff neck |  |  |  |  |  |
| No | 111 | (86.7) | 26 | (86.7) | 1.000 |
| Yes | 17 | (13.3) | 4 | (13.3) |  |
| Dizziness |  |  |  |  |  |
| No | 91 | (71.1) | 17 | (56.7) | 0.126 |
| Yes | 37 | (28.9) | 13 | (43.3) |  |
| Disorientation |  |  |  |  |  |
| No | 108 | (84.4) | 27 | (90.0) | 0.571 |
| Yes | 20 | (15.6) | 3 | (10.0) |  |
| Abdominal pain |  |  |  |  |  |
| No | 88 | (68.8) | 28 | (93.3) | 0.006** |
| Yes | 40 | (31.2) | 2 | (6.7) |  |
| Nose bleed |  |  |  |  |  |
| No | 125 | (97.7) | 30 | (100.0) | 1.000 |
| Yes | 3 | (2.3) | 0 | (0.0) |  |
| Gum bleed |  |  |  |  |  |
| No | 122 | (95.3) | 30 | (100.0) | 0.596 |
| Yes | 6 | (4.7) | 0 | (0.0) |  |
| Abnormal vaginal bleeding |  |  |  |  |  |
| No | 122 | (95.3) | 28 | (93.3) | 0.648 |
| Yes | 6 | (4.7) | 2 | (6.7) |  |
| Blood in urine |  |  |  |  |  |
| No | 124 | (96.9) | 28 | (93.3) | 0.319 |
| Yes | 4 | (3.1) | 2 | (6.7) |  |
| Bruising |  |  |  |  |  |
| No | 126 | (98.4) | 30 | (100.0) | 1.000 |
| Yes | 2 | (1.6) | 0 | (0.0) |  |
| Blood in stool |  |  |  |  |  |
| No | 125 | (97.7) | 30 | (100.0) | 1.000 |
| Yes | 3 | (2.3) | 0 | (0.0) |  |
| Any haemorrhagic manifestation^b^ |  |  |  |  |  |
| No | 105 | (82.0) | 27 | (90.0) | 0.414 |
| Yes | 23 | (18.0) | 3 | (10.0) |  |
|  | Mean | (s.d.)à | Mean | (s.d.)à | p-value |
| Days post onset of illness | 3.66 | (2.00) | 2.80 | (1.83) | 0.032* |
| Days post onset of fever | 3.33 | (1.96) | 2.39 | (1.77) | 0.021* |
| Temperature (C) | 37.79 | (0.83) | 37.87 | (0.64) | 0.640 |
| White blood cell count (10^3^/ul) | 8.36 | (4.85) | 6.52 | (3.25) | 0.016* |
| Haematocrit (%) | 43.81 | (23.70) | 39.00 | (7.99) | 0.276 |
| Platelet count (10^3^/ul) | 213.20 | (100.86) | 234.60 | (72.48) | 0.277 |

^ⱡ^ Defined as a positive result on a Real-time Polymerase Chain Reaction test

^†^ *p*<0.10; ^*^*p*<0.05; ^**^*p*<0.01

^‡^ Standard deviation

^a^ Includes: single, divorced and widower

^b^ Includes: nose bleed, gum bleed, abnormal vaginal bleeding, blood in urine, bruising or blood in stool
