# Supplementary material for: Molecular Characterisation of Chikungunya Virus Infections in Trinidad and Comparison of Clinical and Laboratory Features with Dengue and Other Acute Febrile Cases
Source: PLoS Negl Trop Dis. 2015 Nov 18;9(11):e0004199. doi: 10.1371/journal.pntd.0004199 (PMC4651505; doi:10.1371/journal.pntd.0004199)
Supplement: S5 Table — (DOCX) [file pntd.0004199.s006.docx]

Supplementary Table 5 - Bivariable associations of patients of the Adult Primary Care Facility of the Eric Williams Medical Sciences Complex, Trinidad & Tobago, (Dec 2013–Nov 2014) with having a confirmed infection^ⱡ^ with either any Dengue virus serotype (DENV) or Chikungunya virus (CHIKV)

|  | DENV Status | | CHIKV Status | |  |
| --- | --- | --- | --- | --- | --- |
|  | n | (%) | n | (%) | *p*-value |
| Demographics |  |  |  |  |  |
| Age, years (categorised on median) |  |  |  |  |  |
| <32 | 5 | (62.5) | 11 | (36.7) | 0.243 |
| >32 | 3 | (37.5) | 19 | (63.3) |  |
| Sex |  |  |  |  |  |
| Male | 6 | (75.0) | 11 | (36.7) | 0.107 |
| Female | 2 | (25.0) | 19 | (63.3) |  |
| Ethnicity |  |  |  |  |  |
| Afro-Trinidadian | 0 | (0.0) | 8 | (36.4) | 0.097 |
| Indo-Trinidadian | 1 | (16.7) | 5 | (22.7) |  |
| Mixed | 4 | (66.7) | 5 | (22.7) |  |
| Other | 1 | (16.7) | 4 | (18.2) |  |
| Marital status |  |  |  |  |  |
| Not married^a^ | 3 | (37.5) | 7 | (26.9) | 0.666 |
| Married (including common-law) | 5 | (62.5) | 19 | (73.1) |  |
| Education level |  |  |  |  |  |
| >Secondary school | 3 | (42.9) | 7 | (29.2) | 0.652 |
| <Secondary school | 4 | (57.1) | 17 | (70.8) |  |
| Employment status |  |  |  |  |  |
| Unemployed | 1 | (12.5) | 10 | (35.7) | 0.388 |
| Employed | 7 | (87.5) | 18 | (64.3) |  |
| Income stability |  |  |  |  |  |
| Not daily paid | 7 | (100.0) | 17 | (100.00) | - |
| Daily paid | 0 | (0.0) | 0 | (0.0) |  |
| Nationality |  |  |  |  |  |
| Not Trinidadian or Tobagonian | 1 | (12.5) | 10 | (33.3) | 0.395 |
| Trinidadian or Tobagonian | 7 | (87.5) | 20 | (66.7) |  |
| Risk factors |  |  |  |  |  |
| Travelled outside Trinidad in the 2 weeks prior to interview |  |  |  |  |  |
| No | 6 | (85.7) | 25 | (83.3) | 1.000 |
| Yes | 1 | (14.3) | 5 | (16.7) |  |
| Visited a forested area in Trinidad or Tobago in the 2 weeks prior to interview |  |  |  |  |  |
| No | 4 | (57.1) | 26 | (89.7) | 0.073 |
| Yes | 3 | (42.9) | 3 | (10.3) |  |
| Contact with a wild animal in the 2 weeks prior to interview |  |  |  |  |  |
| No | 8 | (100.0) | 29 | (96.7) | 1.000 |
| Yes | 0 | (0.0) | 1 | (3.3) |  |
| Contact with livestock in the 2 weeks prior to interview |  |  |  |  |  |
| No | 8 | (100.0) | 28 | (93.3) | 1.000 |
| Yes | 0 | (0.0) | 2 | (6.7) |  |
| Contact with a companion animal in the 2 weeks prior to interview |  |  |  |  |  |
| No | 7 | (87.5) | 20 | (66.7) | 0.395 |
| Yes | 1 | (12.5) | 10 | (33.3) |  |
| Previous dengue |  |  |  |  |  |
| No | 4 | (57.1) | 27 | (93.1) | 0.040* |
| Yes | 3 | (42.9) | 2 | (6.9) |  |
| Febrile household member in the 2 weeks prior to interview |  |  |  |  |  |
| No | 5 | (71.4) | 20 | (71.4) | 1.000 |
| Yes | 2 | (28.6) | 8 | (28.6) |  |
| Household member diagnosed with dengue in the 2 weeks prior to interview |  |  |  |  |  |
| No | 7 | (100.0) | 26 | (89.7) | 1.000 |
| Yes | 0 | (0.0) | 3 | (10.3) |  |
| History of mosquito bites at home |  |  |  |  |  |
| No | 1 | (14.3) | 5 | (16.7) | 1.000 |
| Yes | 6 | (85.7) | 25 | (83.3) |  |
| Screened windows at home |  |  |  |  |  |
| No | 4 | (57.1) | 25 | (83.3) | 0.156 |
| Yes | 3 | (42.9) | 5 | (16.7) |  |
| Storage of water at home |  |  |  |  |  |
| No | 5 | (71.4) | 10 | (33.3) | 0.095 |
| Yes | 2 | (28.6) | 20 | (66.7) |  |
| Bushy or unkempt areas around home |  |  |  |  |  |
| No | 5 | (71.4) | 15 | (50.0) | 0.416 |
| Yes | 2 | (28.6) | 15 | (50.0) |  |
| Coworker diagnosed with dengue in the 2 weeks prior to interview |  |  |  |  |  |
| No | 6 | (100.0) | 17 | (81.0) | 0.545 |
| Yes | 0 | (100.0) | 4 | (19.0) |  |
| Clinical factors |  |  |  |  |  |
| History of yellow fever virus vaccination |  |  |  |  |  |
| No | 2 | (25.0) | 21 | (70.0) | 0.039* |
| Yes | 6 | (75.0) | 9 | (30.0) |  |
| History of MMR vaccination |  |  |  |  |  |
| No | 2 | (25.0) | 22 | (73.3) | 0.034* |
| Yes | 6 | (75.0) | 8 | (26.7) |  |
| History of Hepatitis B vaccination |  |  |  |  |  |
| No | 5 | (62.5) | 23 | (76.7) | 0.411 |
| Yes | 3 | (37.5) | 7 | (23.3) |  |
| History of other vaccination |  |  |  |  |  |
| No | 8 | (100.0) | 25 | (83.3) | 0.563 |
| Yes | 0 | (0.0) | 5 | (16.7) |  |
| History of bleeding disorder |  |  |  |  |  |
| No | 7 | (100.0) | 28 | (96.6) | 1.000 |
| Yes | 0 | (0.0) | 1 | (3.4) |  |
| Admitted to hospital |  |  |  |  |  |
| No | 1 | (33.3) | 1 | (50.0) | 1.000 |
| Yes | 2 | (66.7) | 1 | (50.0) |  |
| Headache |  |  |  |  |  |
| No | 1 | (12.5) | 7 | (23.3) | 0.660 |
| Yes | 7 | (87.5) | 23 | (76.7) |  |
| Muscle pain |  |  |  |  |  |
| No | 3 | (37.5) | 9 | (30.0) | 0.689 |
| Yes | 5 | (62.5) | 21 | (70.0) |  |
| Joint pain |  |  |  |  |  |
| No | 4 | (50.0) | 5 | (16.7) | 0.071 |
| Yes | 4 | (50.0) | 25 | (83.3) |  |
| Back pain |  |  |  |  |  |
| No | 7 | (87.5) | 30 | (100.0) | 0.211 |
| Yes | 1 | (12.5) | 0 | (0.0) |  |
| Rash |  |  |  |  |  |
| No | 8 | (100.0) | 20 | (66.7) | 0.082 |
| Yes | 0 | (0.0) | 10 | (33.3) |  |
| Fatigue |  |  |  |  |  |
| No | 7 | (87.5) | 15 | (50.0) | 0.106 |
| Yes | 1 | (12.5) | 15 | (50.0) |  |
| Eye pain |  |  |  |  |  |
| No | 3 | (37.5) | 18 | (60.0) | 0.426 |
| Yes | 5 | (62.5) | 12 | (40.0) |  |
| Cough |  |  |  |  |  |
| No | 5 | (62.5) | 23 | (76.7) | 0.411 |
| Yes | 3 | (37.5) | 7 | (23.3) |  |
| Nausea |  |  |  |  |  |
| No | 4 | (50.0) | 24 | (80.0) | 0.170 |
| Yes | 4 | (50.0) | 6 | (20.0) |  |
| Vomiting |  |  |  |  |  |
| No | 4 | (50.0) | 22 | (73.3) | 0.232 |
| Yes | 4 | (50.0) | 8 | (26.7) |  |
| Diarrhoea |  |  |  |  |  |
| No | 6 | (75.0) | 25 | (83.3) | 0.624 |
| Yes | 2 | (25.0) | 5 | (16.7) |  |
| Sore throat |  |  |  |  |  |
| No | 7 | (87.5) | 29 | (96.7) | 0.381 |
| Yes | 1 | (12.5) | 1 | (3.3) |  |
| Weakness |  |  |  |  |  |
| No | 3 | (37.5) | 9 | (30.0) | 0.689 |
| Yes | 5 | (62.5) | 21 | (70.0) |  |
| Stiff neck |  |  |  |  |  |
| No | 7 | (87.5) | 26 | (86.7) | 1.000 |
| Yes | 1 | (12.5) | 4 | (13.3) |  |
| Dizziness |  |  |  |  |  |
| No | 6 | (75.0) | 17 | (56.7) | 0.440 |
| Yes | 2 | (25.0) | 13 | (43.3) |  |
| Disorientation |  |  |  |  |  |
| No | 8 | (100.0) | 27 | (90.0) | 1.000 |
| Yes | 0 | (0.0) | 3 | (10.0) |  |
| Abdominal pain |  |  |  |  |  |
| No | 5 | (62.5) | 28 | (93.3) | 0.053 |
| Yes | 3 | (37.5) | 2 | (6.7) |  |
| Nose bleed |  |  |  |  |  |
| No | 8 | (100.0) | 30 | (100.0) | - |
| Yes | 0 | (0.0) | 0 | (0.0) |  |
| Gum bleed |  |  |  |  |  |
| No | 8 | (100.0) | 30 | (100.0) | - |
| Yes | 0 | (0.0) | 0 | (0.0) |  |
| Abnormal vaginal bleeding |  |  |  |  |  |
| No | 8 | (100.0) | 28 | (93.3) | 1.000 |
| Yes | 0 | (0.0) | 2 | (6.7) |  |
| Blood in urine |  |  |  |  |  |
| No | 8 | (100.0) | 28 | (93.3) | 1.000 |
| Yes | 0 | (0.0) | 2 | (6.7) |  |
| Bruising |  |  |  |  |  |
| No | 8 | (100.0) | 30 | (100.0) | - |
| Yes | 0 | (0.0) | 0 | (0.0) |  |
| Blood in stool |  |  |  |  |  |
| No | 8 | (100.0) | 30 | (100.0) | - |
| Yes | 0 | (0.0) | 0 | (0.0) |  |
| Any haemorrhagic manifestation^b^ |  |  |  |  |  |
| No | 8 | (100.0) | 27 | (90.0) | 1.000 |
| Yes | 0 | (0.0) | 3 | (10.0) |  |
|  | Median | Min-Maxà | Median | Min-Maxà | p-value |
| Days post onset of illness | 3.50 | 1.0-5.0 | 2.50 | 0.0-7.0 | 0.490 |
| Days post onset of fever | 2.50 | 1.0-5.0 | 2.00 | 0.0-6.0 | 0.484 |
| Temperature (C) | 37.00 | 36.7-39.3 | 38.0 | 37.0-39.0 | 0.288 |
| White blood cell count (10^3^/ul) | 3.50 | 1.7-6.3 | 6.00 | 2.7-17.6 | 0.028* |
| Haematocrit (%) | 43.10 | 37.9-48.8 | 39.85 | 12.9-51.4 | 0.092 |
| Platelet count (10^3^/l) | 147.00 | 51.0-294.0 | 239.0 | 83.0-375.0 | 0.022* |
