# Supplementary material for: Molecular Characterisation of Chikungunya Virus Infections in Trinidad and Comparison of Clinical and Laboratory Features with Dengue and Other Acute Febrile Cases
Source: PLoS Negl Trop Dis. 2015 Nov 18;9(11):e0004199. doi: 10.1371/journal.pntd.0004199 (PMC4651505; doi:10.1371/journal.pntd.0004199)
Supplement: S6 Table — (DOCX) [file pntd.0004199.s007.docx]

Supplementary Table 6 - Percentage nucleotide and amino acid identities across the open reading frame of CHIKV

|  | **VE53_20** | **VE54_19** | **VE54_20** | **VE55_4** | **VE56_9** | **VE56_13** | **VE56_20** | **VE57_2** | **KJ451624** |
| --- | --- | --- | --- | --- | --- | --- | --- | --- | --- |
| **VE53_20** |  | 99.93 (99.87) | 99.93 (99.90) | 99.93 (99.85) | 99.91 (99.90) | 99.92 (99.95) | 99.93 (99.87) | 99.90 (99.82) | 99.94 (99.87) |
| **VE54_19** | 99.93 (99.87) |  | 99.96 (99.98) | 99.96 (99.93) | 99.93 (99.98) | 99.94 (99.93) | 99.95 (99.95) | 99.93 (99.90) | 99.97 (99.95) |
| **VE54_20** | 99.93 (99.90) | 99.96 (99.98) |  | 99.97 (99.95) | 99.98 (100) | 99.98 (99.95) | 99.99 (99.98) | 99.93 (99.93) | 99.98 (99.98) |
| **VE55_4** | 99.93 (99.85) | 99.96 (99.93) | 99.97 (99.95) |  | 99.94 (99.95) | 99.95 (99.90) | 99.96 (99.93) | 99.97 (99.98) | 99.98 (99.93) |
| **VE56_9** | 99.91 (99.90) | 99.93 (99.98) | 99.98 (100) | 99.94 (99.95) |  | 99.96 (99.95) | 99.97 (99.98) | 99.91 (99.93) | 99.95 (99.98) |
| **VE56_13** | 99.92 (99.85) | 99.94 (99.93) | 99.98 (99.95) | 99.95 (99.90) | 99.96 (99.95) |  | 99.98 (99.93) | 99.92 (99.87) | 99.96 (99.93) |
| **VE56_20** | 99.93 (99.87) | 99.95 (99.95) | 99.99 (99.98) | 99.96 (99.93) | 99.97 (99.98) | 99.98 (99.93) |  | 99.93 (99.90) | 99.97 (99.95) |
| **VE57_2** | 99.90 (99.82) | 99.93 (99.90) | 99.93 (99.93) | 99.97 (99.98) | 99.91 (99.93) | 99.92 (99.87) | 99.93 (99.90) |  | 99.94 (99.90) |
| **KJ451624** | 99.94 (99.87) | 99.97 (99.95) | 99.98 (99.98) | 99.98 (99.93) | 99.95 (99.98) | 99.96 (99.93) | 99.97 (99.95) | 99.94 (99.90) |  |

sequences from Trinidad and the British Virgin Islands
